# Associations between metal concentrations in whole blood and placenta previa and placenta accreta: the Japan Environment and Children’s Study (JECS)

**DOI:** 10.1186/s12199-019-0795-7

**Published:** 2019-06-07

**Authors:** Mayumi Tsuji, Eiji Shibata, David J. Askew, Seiichi Morokuma, Yukiyo Aiko, Ayako Senju, Shunsuke Araki, Masafumi Sanefuji, Yasuhiro Ishihara, Rie Tanaka, Koichi Kusuhara, Toshihiro Kawamoto, Japan Environment and Children’s Study Group, Toshihiro Kawamoto, Hirohisa Saito, Reiko Kishi, Nobuo Yaegashi, Koichi Hashimoto, Chisato Mori, Shuichi Ito, Zentaro Yamagata, Hidekuni Inadera, Michihiro Kamijima, Takeo Nakayama, Hiroyasu Iso, Masayuki Shima, Yasuaki Hirooka, Narufumi Suganuma, Koichi Kusuhara, Takahiko Katoh

**Affiliations:** 1 Department of Environmental Health, University of Occupational and Environmental Health, Kitakyushu, Japan; 2 Department of Obstetrics and Gynecology, School of Medicine, University of Occupational and Environmental Health, Kitakyushu, Japan; 3 Research Center for Environmental and Developmental Medical Sciences, Kyushu University, Fukuoka, Japan; 4 Japan Environment and Children’s Study, University of Occupational and Environmental Health Subunit Center, University of Occupational and Environmental Health, Kitakyushu, Fukuoka, Japan; 5 Department of Pediatrics, School of Medicine, University of Occupational and Environmental Health, Kitakyushu, Japan; 6 Laboratory of Molecular Brain Science, Graduate School of Integrated Arts and Sciences, Hiroshima University, Higashi-Hiroshima, Japan

**Keywords:** Metal concentration, Placenta previa, Placenta accreta, Pregnancy

## Abstract

**Background:**

Placenta previa and placenta accreta associate with high morbidity and mortality for both mothers and fetus. Metal exposure may have relationships with placenta previa and placenta accreta. This study analyzed the associations between maternal metal (cadmium [Cd], lead [Pb], mercury [Hg], selenium [Se], and manganese [Mn]) concentrations and placenta previa and placenta accreta.

**Methods:**

We recruited 17,414 women with singleton pregnancies. Data from a self-administered questionnaire regarding the first trimester and medical records after delivery were analyzed. Maternal blood samples were collected to measure metal concentrations. The subjects were classified into four quartiles (Q1, Q2, Q3, and Q4) according to metal concentrations.

**Results:**

The odds ratio for placenta previa was significantly higher among subjects with Q4 Cd than those with Q1 Cd. The odds ratio for placenta previa was significantly higher for subjects with Q2 Pb than those with Q1 Pb.

**Conclusion:**

Participants with placenta previa had higher Cd concentrations. However, this study was cross-sectional and lacked important information related to Cd concentration, such as detailed smoking habits and sources of Cd intake. In addition, the subjects in this study comprised ordinary pregnant Japanese women, and it was impossible to observe the relationship between a wide range of Cd exposure and placenta previa. Therefore, epidemiological and experimental studies are warranted to verify the relationship between Cd exposure and pregnancy abnormalities.

**Electronic supplementary material:**

The online version of this article (10.1186/s12199-019-0795-7) contains supplementary material, which is available to authorized users.

## Introduction

Placenta previa is a condition in which the placenta is attached to the lower uterine segment and completely or partially covers the internal cervix [1]. When chorionic villi abnormally invade the myometrium, placenta accreta occurs [2]. Currently, reported rates of placenta previa are between 0.3 and 0.8% [3, 4]. The placenta accreta rate varies because of differences in subjects; however, it is 0.4% in Japan [5, 6]. Placenta previa and placenta accreta are related: 9.3% of women with placenta previa have placenta accreta [2]. Both cases present risks to the mother and fetus. For the mother, affected pregnancies are associated with excessive hemorrhaging, damage to surrounding organs, and death. The fetus may experience preterm delivery, may be small for gestational age, or may have congenital defects [7, 8].

Successful implantation requires the following complex mechanisms: the fertilized egg/blastocyst migrates from the oviduct to the uterine cavity and orients in appropriate regions of the uterus (migration); the trophoblast attaches to the uterine epithelium (attachment); the trophoblast attaches firmly to the uterine epithelium (adhesion); the trophoblast penetrates the uterine epithelium (penetration); and the trophoblast invades the uterine endometrium (invasion) [9–11]. Placenta previa and placenta accreta share overlapping risk factors, many of which are associated with disruption of the normal uterine endometrium. These risks include previous cesarean deliveries, manual removal of the placenta, or other gynecological surgeries that result in scarring and are likely to lead to inappropriate attachment during implantation [12, 13].

Environmental exposure to smoking and air pollution has also been reported as a risk factor for placenta previa and placenta accreta [14, 15]. Smoking influences the maternal immune response and inflammatory response during pregnancy [16, 17]. Inflammation leads to female genital tract damage, including damage to the endometrial and myometrial epithelium [18]. Therefore, smoking may induce inappropriate attachment and lead to placenta previa and placenta accreta. Another important factor during the implantation process is angiogenesis [19, 20]. Placental vascularization has been found to be significantly decreased in smoke-exposed placentas [21]. Cigarettes contain many harmful substances, including heavy metals [22], and current and former smoking has been related to high cadmium (Cd) and lead (Pb) levels in uterus tissues [23]. Exposure to heavy metals, especially Cd and Pb, impacts the female reproductive system [24]. Furthermore, Cd, Pb, and mercury (Hg) might affect endometrial angiogenesis [25, 26]. However, no studies have examined the relationship between metal exposure and placenta previa and placenta accrete directly.

Selenium (Se) and manganese (Mn) are essential elements which have been related to blastocyst quality and implantation [27–29]. However, there is no research to investigate the relationship between these metals and placenta previa and placenta accrete.

Therefore, the purpose of this study was to examine the relationship between metal exposure and placental previa and placenta accreta using the Japan Environment and Children’s Study (JECS), which is a large cohort study.

## Methods

### Study subjects

During this study, our target subjects were pregnant women participating in the JECS. They were recruited during early pregnancy at obstetric facilities and/or local government offices [30] in 15 regions across a wide geographical area in Japan between January 2011 and March 2014.

**Fig. 1:**
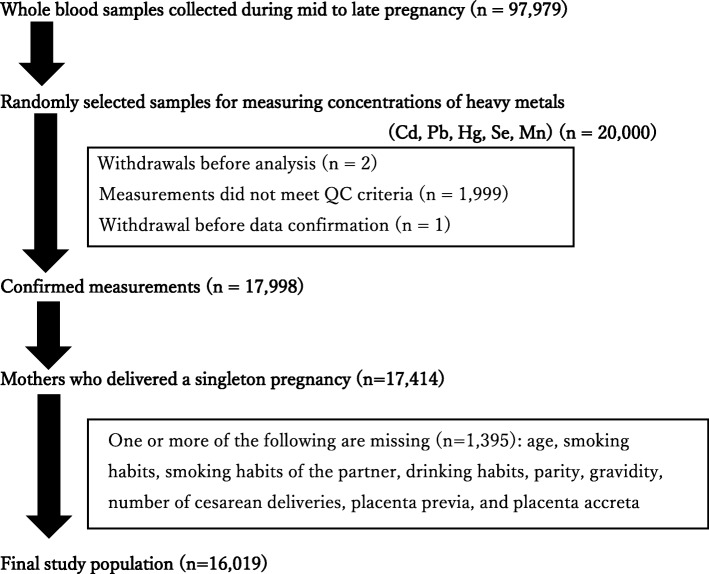
Flow chart of the study subjects. Of the samples collected from the 97,979 women during mid to late pregnancy, 20,000 whole blood samples were randomly selected. To measure metal concentrations, 17,998 blood samples of women who met the QC criteria were used. Of these 17,998, 17,414 who delivered singleton pregnancies were selected. After excluding women with missing data (*N* = 1395), the final study population comprised of 16,019 women. Cd, cadmium; Pb, lead; Hg, mercury; Se, selenium; Mn, manganese; QC, quality control

Of the 17,998 women whose metal concentrations were measured, 17,414 had singleton pregnancies. After excluding women with missing data (*N* = 1395), 16,019 women were selected for analysis (Fig. 1).

### Questionnaires

Self-administered questionnaires were provided at prenatal examinations or by mail to the women twice during pregnancy (during the first trimester [T1] and the second/third trimester [T2]). Data from the questionnaire provided during T1 were used in this study. Medical record transcription was performed by physicians, midwives/nurses, or research coordinators after delivery [30–32]. Data for those with and without placenta previa and placenta accreta were obtained from medical records. The present study was based on the jecs-ag-ai-20,160,424 data set, which was released in June 2016 and revised in October 2016.

### Blood collection

Metal concentrations were measured in T2 blood samples comprising 33 mL of blood collected from each pregnant mother. The present study was based on the jecs-mtl-ai-20,170,403 data set, which was released in April 2017.

### Measurements of metal concentrations in blood

We measured the metal concentrations in the blood according to our previous report [33]. Briefly, blood samples with sodium ethylenediaminetetraacetic acid (EDTA) were transferred to a central laboratory and stored at − 80 °C before use. A standard solution comprising all target elements except Hg was prepared in 0.14 M nitric acid. An Hg standard solution was produced using a solution made from 0.056 M nitric acid, 0.5% *w*/*v* EDTA, and 1% *v*/*v* tetramethylammonium hydroxide (TMAH). The final concentrations of Hg, Pb, Cd, Mn, and Se in the standard solution were 200, 200, 20, 600, and 2000 ng/g, respectively. An internal standard (yttrium, indium, and thallium at 250 ng/g) was prepared using 0.14 M nitric acid. Blood samples (200 μL) were diluted (1:19) with the dilution solution (2% *v*/*v* butan-1-ol, 0.1% TMAH, 0.05% *w*/*v* polyoxyethlene octylphenyl ether, and 0.05% *w*/*v* H4EDTA) [10] and vortex-mixed before the inductively coupled plasma mass spectrometry (ICP-MS) analysis.

ICP-MS measurements of metals in the blood were performed using Agilent 7700 ICP-MS (Agilent Technologies, Tokyo, Japan). Method detection limits for each analyte were calculated according to the method described previously [34]. The intensity of Pb isotypes was the summation of that of ^206^Pb, ^207^Pb, and ^208^Pb. To correct the spectral overlap from molybdenum oxide (95Mo16O), the intensity of ^111^Cd was calculated using the following equation:

$$ \left[\mathrm{Cd}\right]=\left[m/z\ 111\ \mathrm{intensity}\right]-\left[m/z\ 95\ \mathrm{intensity}\right]\times \left[95\mathrm{Mo}16\mathrm{O}\ \mathrm{generation}\ \mathrm{rate}\right] $$

The 95Mo16O generation rate was derived from the following equation:

$$ \left[95\mathrm{Mo}16\mathrm{O}\ \mathrm{generation}\ \mathrm{rate}\right]=\left[\mathrm{the}\ \mathrm{intensity}\ \mathrm{of}\ m/z\ 111\ \mathrm{when}\ 100\;\mathrm{ppb}\ 95\mathrm{Mo}\ \mathrm{standard}\ \mathrm{solution}\ \mathrm{was}\ \mathrm{analyzed}\right]/\left[\mathrm{the}\ \mathrm{intensity}\ \mathrm{of}\ m/z\ 96\ \mathrm{when}\ 100\;\mathrm{ppb}\ 95\mathrm{Mo}\ \mathrm{standard}\ \mathrm{solution}\ \mathrm{was}\ \mathrm{analyzed}\right] $$

### Statistical methods

Two-group comparisons were performed using the Mann-Whitney *U* test and multivariable logistic regression analyses. The *p* values of the multivariable logistic regression analysis were calculated after adjusting for age, smoking, smoking habits of the partner, drinking habits, gravidity, parity, number of cesarean deliveries, and geographic region [35, 36]. Placenta previa was added as a covariate when comparisons were performed with or without placenta accreta. Subjects were divided equally into quartiles depending on individual metal concentrations (first quartile [Q1], second quartile [Q2], third quartile [Q3], fourth quartile [Q4]); these quartiles were used in the multivariable logistic regression analysis and trend test.

All analyses were performed using Stata version 14 (Stata Corp., College Station, TX, USA), and statistical significance was assumed when *p* < 0.05 (two-sided).

## Results

Table 1 shows characteristics of the study population. The rates of placenta previa and placenta accreta were 0.5% and 0.2%, respectively, among subjects. Four women had placenta previa and placenta accreta.

**Table 1:** Study population characteristics

	Total (*N* = 16,019)	Previa			Accreta		
		Without (*N* = 15,929)	With (*N* = 90)	*p* value^†^	Without (*N* = 15,980)	With (*N* = 39)	*P* value^†^
Age (years)	31.3 ± 5.0	31.3 ± 5.0	32.9 ± 4.7	0.003	31.3 ± 5.0	34.1 ± 5.2	0.002
Smoking habits							
Never	9194 (57)	9136 (57)	58 (64)		9173 (57)	21 (54)	
Former	6002 (37)	5974 (38)	28 (31)	0.224	5989 (37)	13 (33)	1.000
Current	823 (5)	819 (5)	4 (4)	0.817	818 (5)	5 (13)	0.057
Smoking habits of partner							
Never	4281 (27)	4251 (27)	30 (33)		4265 (27)	16 (41)	
Former	4339 (27)	4314 (27)	25 (28)	0.501	4326 (27)	13 (33)	0.582
Current	7399 (46)	7364 (46)	35 (39)	0.122	7389 (46)	10 (26)	0.013
Drinking habits							
No	14,386 (90)	14,307 (90)	79 (88)		14,355 (90)	31 (79)	
Yes	1633 (10)	1622 (10)	11 (12)	0.485	1625 (10)	8 (21)	0.055
Gravidity							
Primigravida	4758 (30)	4733 (30)	25 (28)		4751 (30)	7 (18)	
Multigravida	11,261 (70)	11,196 (70)	65 (72)	0.730	11,229 (70)	32 (82)	0.117
Parity							
Nulliparous	6385 (40)	6348 (40)	37 (41)		6372 (40)	13 (33)	
Multiparous	9634 (60)	9581 (60)	53 (59)	0.829	9608 (60)	26 (67)	0.513
Number of cesarean deliveries							
0	14,576 (91)	14,496 (91)	80 (89)		14,542 (91)	34 (87)	
1	1111 (7)	1106 (7)	5 (6)	0.833	1108 (7)	3 (8)	0.744
2	305 (2)	300 (2)	5 (6)	0.030	303 (2)	2 (5)	0.168
≥ 3	27 (0)	27 (0)	0 (0)	1.000	27 (0)	0 (0)	1.000

Values are mean ± SD or number (%)

†*p* values for age were obtained using Welch’s *t* test. *p* values for other factors were obtained using Fisher’s exact test

Table 2 shows the distribution of metal concentrations by smoking habits. Cd and Pb concentrations of current smokers were high in our study (Cd; *p* < 0.001, Pb; *p* < 0.001 by analysis of variance [ANOVA]). In contrast, Hg and Mn concentrations of current smokers were low in our study (Hg; *p* < 0.001, Mn; *p* < 0.001 by ANOVA).

**Table 2:** The distribution of metal concentrations by smoking habits

	Median (ng/g) (25th and 75th percentiles)^a^				*p* value*
	Total (*N* = 16,019)	Smoking habits			
		Never (*N* = 9194)	Former (*N* = 6002)	Current (*N* = 823)	
Cd	0.66 (0.50, 0.91)	0.64 (0.48, 0.86)	0.66 (0.50, 0.90)	1.07 (0.78, 1.44)	< 0.001
Pb	5.96 (4.80, 7.45)	5.81 (4.69, 7.24)	6.08 (4.93, 7.61)	6.92 (5.56, 8.74)	< 0.001
Hg	3.65 (2.57, 5.16)	3.75 (2.65, 5.27)	3.53 (2.51, 5.00)	3.40 (2.35, 5.02)	< 0.001
Se	169 (158, 183)	169 (157, 183)	170 (158, 183)	170 (158, 184)	0.339
Mn	15.3 (12.6, 18.7)	15.5 (12.8, 18.8)	15.2 (12.5, 18.6)	14.8 (12.3, 17.9)	< 0.001

*p values were obtained using ANOVA

^a^Natural log-transformed variables were used for ANOVA

**Fig. 2:**
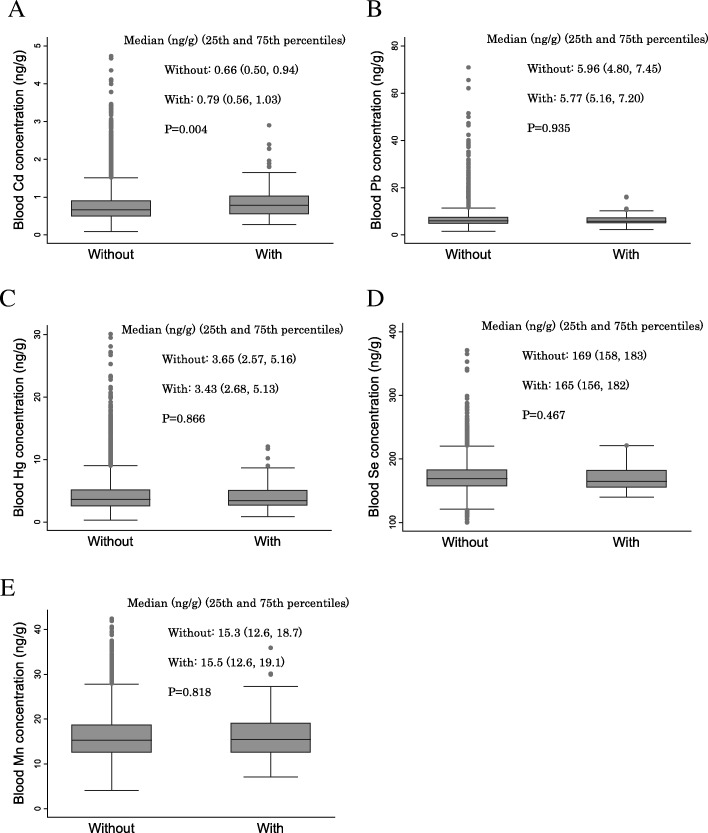
Relationships between metal concentrations in the blood and placenta previa. The relationships between metal concentrations in the second/third trimester (T2) blood and placenta previa are shown. A box plot displays a box bordered at the 25th and 75th percentiles of the variable on the y-axis with a median line at the 50th percentile of each metal concentration. *p* values were calculated using the Mann-Whitney *U* test. **a** Relationship between Cd concentration and placenta previa. **b** Relationship between Pb concentration and placenta previa. **c** Relationship between Hg concentration and placenta previa. **d** Relationship between Se concentration and placenta previa. **e** Relationship between Mn concentration and placenta previa. Cd, cadmium; Pb, lead; Hg, mercury; Se, selenium; Mn, manganese

Figure 2 shows the relationship between metal concentrations and placenta previa. There was a significant difference in Cd concentrations for the two placenta previa groups (median [ng/g]: without, 0.66; with, 0.79; *P* = 0.004). There were no significant differences between other metals and placenta previa.

**Fig. 3:**
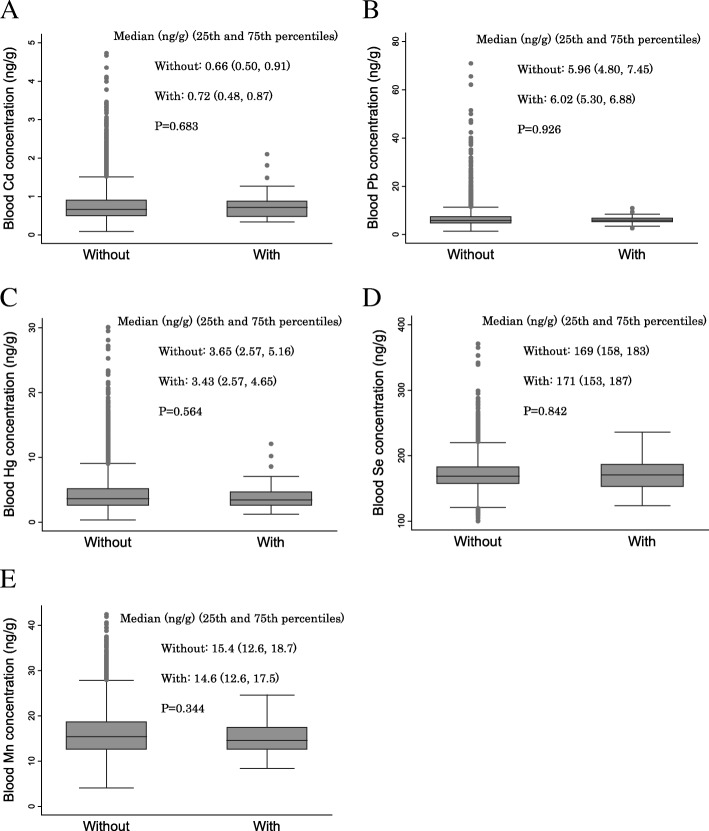
Relationships between metal concentrations in the blood and placenta accreta. The relationships between metal concentrations in the second/third trimester (T2) blood and placenta accreta are shown. A box plot displays a box bordered at the 25th and 75th percentiles of the variable on the y-axis with a median line at the 50th percentile of each metal concentration. *p* values were calculated using the Mann-Whitney *U* test. **a** Relationship between Cd concentration and placenta accreta. **b** Relationship between Pb concentration and placenta accreta. **c** Relationship between Hg concentration and placenta accreta. **d** Relationship between Se concentration and placenta accreta. **e** Relationship between Mn concentration and placenta accreta. Cd, cadmium; Pb, lead; Hg, mercury; Se, selenium; Mn, manganese

Figure 3 shows the relationship between metal concentrations and placenta accreta. There were no significant differences between all metals and placenta accreta.

To further assess the association between levels of metal concentrations and placenta previa and placenta accreta, we performed multivariable logistic regression analyses by using the quartile variables of metal concentrations. The odds ratio (OR) for placenta previa was significantly higher among subjects with Q4 Cd than those with Q1 Cd (OR, 2.06; 95% confidence interval [CI], 1.07, 3.98; *p* = 0.031). Further, the OR for placenta previa was significantly higher among subjects with Q2 Pb than those with Q1 Pb (OR, 2.59; 95% CI, 1.40, 4.80; *p* = 0.003). There was a significant relationship between placenta previa and Pb concentrations (P for trend = 0.007). There was no significant relationship between placenta accreta and metal concentrations (Table 3).

**Table 3:** Results of multivariable analysis regarding the relationship between quartile concentrations of metals, placenta previa, and placenta accreta

Quartile concentration of metals (ng/g)	Previa				Accreta			
	Without (*N* = 15,929)	With (*N* = 90)	OR (95% CI)	*p* value*	Without (*N* = 15,980)	With (*N* = 39)	OR (95% CI)	*p* value **
Cd								
Q1 (≤ 0.496)	3967	14	1.00 (referent)		3971	10	1.00 (referent)	
Q2 (0.497–0.661)	3999	20	1.37 (0.69–2.72)	0.375	4012	7	0.55 (0.21–1.46)	0.234
Q3 (0.662–0.904)	3982	25	1.67 (0.86–3.26)	0.129	3993	14	1.01 (0.44–2.33)	0.981
Q4 (≥ 0.905)	3981	31	2.06 (1.07–3.98)	0.031	4004	8	0.46 (0.17–1.22)	0.120
			P for trend =	0.146			P for trend =	0.205
Pb								
Q1 (≤4.79)	3969	14	1.00 (referent)		3976	7	1.00 (referent)	
Q2 (4.80–5.95)	3987	37	2.59 (1.40–4.80)	0.003	4012	12	1.46 (0.57–3.76)	0.429
Q3 (5.96–7.44)	3975	19	1.32 (0.66–2.64)	0.436	3981	13	1.68 (0.66–4.24)	0.276
Q4 (≥ 7.45)	3998	20	1.34 (0.67–2.67)	0.411	4011	7	0.79 (0.27–2.30)	0.667
			P for trend =	0.007			P for trend =	0.345
Hg								
Q1 (≤ 2.56)	3948	20	1.00 (referent)		3959	9	1.00 (referent)	
Q2 (2.57–3.64)	3987	29	1.37 (0.78–2.44)	0.276	4003	13	1.31 (0.55–3.08)	0.540
Q3 (3.65–5.15)	4004	19	0.89 (0.47–1.67)	0.708	4012	11	1.18 (0.48–2.86)	0.721
Q4 (≥ 5.16)	3990	22	1.03 (0.56–1.90)	0.924	4006	6	0.63 (0.22–1.77)	0.378
			P for trend =	0.477			P for trend =	0.458
Se								
Q1 (≤ 157)	3961	27	1.00 (referent)		3978	10	1.00 (referent)	
Q2 (158–168)	3687	21	0.84 (0.47–1.49)	0.544	3700	8	0.86 (0.34–2.20)	0.756
Q3 (169–182)	4197	21	0.73 (0.41–1.30)	0.284	4209	9	0.83 (0.34–2.07)	0.695
Q4 (≥ 183)	4084	21	0.75 (0.42–1.33)	0.320	4093	12	1.13 (0.48–2.63)	0.784
			P for trend =	0.693			P for trend =	0.901
Mn								
Q1 (≤ 12.5)	3858	21	1.00 (referent)		3871	8	1.00 (referent)	
Q2 (12.6–15.2)	3961	22	1.05 (0.58–1.91)	0.877	3966	17	2.11 (0.91–4.92)	0.083
Q3 (15.3–18.6)	4100	23	1.06 (0.58–1.92)	0.853	4116	7	0.82 (0.31–2.29)	0.710
Q4 (≥ 18.7)	4010	24	1.14 (0.63–2.06)	0.656	4027	7	0.85 (0.31–2.37)	0.758
			P for trend =	0.977			P for trend =	0.080

*p values were obtained from the multivariable logistic regression analysis adjusted for age, smoking, smoking habits of the partner, drinking habits, gravidity, parity, number of cesarean deliveries, and geographic region

**p values were obtained from the multivariable logistic regression analysis adjusted for age, smoking, smoking habits of the partner, drinking habits, gravidity, parity, number of cesarean deliveries, placenta previa, and geographic region

## Discussion

Two significant results were obtained during this research. First, the group with placenta previa had higher Cd concentrations than the group without placenta previa. In addition, subjects with Q4 Cd were at higher OR for placenta previa than those with Q1 Cd. Second, subjects with Q2 Pb were at significantly higher OR for placenta previa than those with Q1 Pb.

Cd and Pb have several exposure sources, such as food and soil [37, 38]. Especially smoking is a major source of Cd and Pb [39]. Other reports have described the relationship between smoking and placenta previa [14, 40]. Indeed, the Cd and Pb concentrations of smokers were high in our study. Therefore, tobacco acts as one of the definite sources of Cd and Pb exposure. However, we categorized the smoking habits for the two groups—never smokers or former/current smokers—and we performed multivariable logistic regression analyses between metal concentrations and placenta previa. In the group of never smokers, the OR of placenta previa was higher among subjects with Q2–Q4 Cd and Pb than those with Q1 (Additional file 1: Table S1). These results show the same trend as seen in Table 3, which involved analysis without dividing the groups into never smokers and former/current smokers. Therefore, the possibility that Cd and Pb are themselves related to placenta previa cannot be discounted.

The median Cd concentration in our study was 0.66 ng/g, which was lower than those reported previously in Japan [41, 42] and low compared to those of residents living in Cd-polluted areas [43]. Regarding Cd, in recent years, it has been reported that exposure to low-dose Cd affects the health of those in non-polluted areas and non-smokers [44]. Therefore, further epidemiological studies and experimental studies involving cell lines and animals are warranted to clarify the relationship between placenta previa and various dose Cd exposure.

The OR for placenta previa was significantly higher among subjects with Q2 Pb than those with Q1 Pb. Women with Q3 Pb group and Q4 Pb group did not demonstrate a significant relationship; however, the OR for placenta previa was higher in those in the Q1 Pb group. It is not clear why Q2 Pb had the highest OR. Therefore, although not in a dose-dependent manner, it cannot be denied that Pb exposure may have some effect on the placenta previa.

Our study has several limitations. First, the source of exposure to Cd in this study was unknown. Smoking is one of the sources of exposure to Cd; however, we had no information regarding the number of cigarettes per day or when subjects quit smoking. Therefore, we could not accurately determine the influence of Cd exposure attributable to smoking. In addition to smoking, there are various other sources of Cd. Among all food sources, rice was the most significant contributor of Cd, followed by vegetables, seaweed, seafood, and millet [45]. Therefore, Cd is absorbed into the human body via these foods. The food intake of some individuals in Japan is regionally dependent. For example, residents in the area along the sea coast have a tendency to eat more seafood, such as shellfish, squid, and crab, which contain much Cd [46, 47]. Therefore, it is necessary to investigate the relationships between the source of Cd exposure, intake amount, blood concentrations, and placenta previa, and to consider the regional characteristics of the food consumed. Second, this study only investigated the relationship between placenta previa and low-dose Cd exposure. We could not clarify which dose of Cd (low or high) had the most influence on placenta previa. Therefore, in the future, it will be important to compare high Cd-polluted areas and low-Cd-polluted areas and high Cd intake groups and low-Cd intake groups. Additional experiments are needed to uncover direct relationships between Cd and placenta previa and/or the mechanism by which Cd can affect placental formation and development. Third, metal concentrations in the placental tissue were not measured in the JECS study. In the future, studies should aim to measure the concentrations of the metal in the placental tissue and to investigate the relationship between these concentrations and placental abnormalities. These studies should help clarify the relationship between metal exposure and placental abnormalities. Fourth, this study was a cross-sectional study using JECS data. Therefore, the causal relationship between Cd exposure and placenta previa could not be considered using only this study. Iron deficiency during pregnancy leads to increased Cd absorption and burden on the body [48]. However, it is unknown whether the increase in Cd absorption caused by iron deficiency affects the onset of placenta previa. To clarify the causal relationship between iron deficiency, Cd absorption, and placenta previa, it is necessary to measure Cd, iron, and ferritin concentrations in the blood throughout pregnancy, especially during the first trimester, which is an important time for normal implantation and placentation [3]. Dietary habits and nutritional status during pregnancy may be related to pregnancy abnormalities [49, 50]; therefore, it is also important to monitor the diet during pregnancy. Furthermore, the JECS study was a cohort study; therefore, it is important to link the relationship between maternal metal exposure, pregnancy abnormalities, and children’s health in the future.

## Conclusion

The group with placenta previa had higher whole blood concentrations of Cd than did the group without placenta previa. However, this study was cross-sectional and did not aim at Cd exposure and its health effects specifically, resulting in a lack of important information regarding Cd concentration, such as detailed smoking habits and sources of Cd intake. In addition, this study was conducted on ordinary Japanese pregnant women, and it was impossible to observe the relationship between a wide range of Cd exposure and placenta previa. Placenta previa involves the risk of excessive hemorrhaging, damage to surrounding organs, and death for the mother. In addition, it can cause preterm birth, small for the gestational age status for the fetus, and congenital defects in the fetus. To prevent placenta previa, maintain the mother’s health, and protect the health of the fetus, epidemiological and experimental studies are warranted to verify the relationship between Cd exposure and pregnancy abnormalities.

## Additional file

Additional file 1: **Table S1.** Results of multivariable analysis for determining the relationship between quartile concentrations of metals and placenta previa in never smokers and former/current smokers. (DOCX 26 kb)

## Data Availability

The data used to derive our conclusions are unsuitable for public deposition due to ethical restrictions and specific legal framework in Japan. It is prohibited by the Act on the Protection of Personal Information (act no. 57 of 30 May 2003, amended on 9 September 2015) to publicly deposit data containing personal information. The Ethical Guidelines for Epidemiological Research enforced by the Japan Ministry of Education, Culture, Sports, Science and Technology and the Ministry of Health, Labor and Welfare also restricts the open sharing of the epidemiologic data. All inquiries about access to data should be sent to jecs-en@nies.go.jp. The person responsible for handling inquiries sent to this e-mail address is Dr. Shoji F. Nakayama, JECS Programme Office, National Institute for Environmental Studies.

## Abbreviations

Cd: Cadmium
CI: Confidence interval
EDTA: Ethylenediaminetetraacetic acid
Hg: Mercury
ICP-MS: Inductively coupled plasma mass spectrometry
Mn: Manganese
OR: Odds ratio
Pb: Lead
Q1: First quartile
Q2: Second quartile
Q3: Third quartile
Q4: Fourth quartile
QC: Quality control
Se: Selenium
T1: The first trimester
T2: The second/third trimester
TMAH: Tetramethylammonium hydroxide
