# Supplementary material for: Associations between metal concentrations in whole blood and placenta previa and placenta accreta: the Japan Environment and Children’s Study (JECS)
Source: Environ Health Prev Med. 2019 Jun 7;24:40. doi: 10.1186/s12199-019-0795-7 (PMC6556030; doi:10.1186/s12199-019-0795-7)
Supplement: Supplementary file 1 — Table S1. Results of multivariable analysis for determining the relationship between quartile concentrations of metals and placenta previa in never smokers and former/current smokers. (DOCX 26 kb) [file 12199_2019_795_MOESM1_ESM.docx]

**Additional file 1: Table S1. Results of multivariable analysis for determining the relationship between quartile concentrations of metals and placenta previa in never- smokers and former-/current- smokers.**

|  | **Never- smokers** | | | | |  | **Former-/current-smokers** | | | |
| --- | --- | --- | --- | --- | --- | --- | --- | --- | --- | --- |
| Quartile concentration of metals (ng/g) | Without (N=9,136) | With (N=58) |  | OR (95% CI) | P value* |  | Without (N=6,793) | With (N=32) | OR (95% CI) | P value* |
| **Cd** |  |  |  |  |  |  |  |  |  |  |
| Q1 (≤0.496) | 2,502 | 8 |  | 1.00 (referent) |  |  | 1,465 | 6 | 1.00 (referent) |  |
| Q2 (0.497-0.661) | 2,347 | 15 |  | 1.88  (0.79-4.47) | 0.151 |  | 1,652 | 5 | 0.71  (0.22-2.35) | 0.577 |
| Q3 (0.662-0.904) | 2,308 | 16 |  | 1.96  (0.83-4.66) | 0.126 |  | 1,674 | 9 | 1.25  (0.44-3.59) | 0.672 |
| Q4 (≥0.905) | 1,979 | 19 |  | 2.54  (1.08-5.98) | 0.033 |  | 2,002 | 12 | 1.37  (0.48-3.89) | 0.672 |
|  |  |  |  | P for trend = | 0.164 |  |  |  | P for trend = | 0.634 |
| **Pb** |  |  |  |  |  |  |  |  |  |  |
| Q1 (≤4.79) | 2,512 | 10 |  | 1.00 (referent) |  |  | 1,457 | 4 | 1.00 (referent) |  |
| Q2 (4.80-5.95) | 2,346 | 25 |  | 2.58  (1.24-5.40) | 0.012 |  | 1,641 | 12 | 2.65  (0.85-8.25) | 0.093 |
| Q3 (5.96-7.44) | 2,230 | 10 |  | 1.08  (0.45-2.61) | 0.860 |  | 1,745 | 9 | 1.79  (0.55-5.86) | 0.334 |
| Q4 (≥7.45) | 2,048 | 13 |  | 1.45  (0.63-3.34) | 0.380 |  | 1,950 | 7 | 1.18  (0.34-4.10) | 0.792 |
|  |  |  |  | P for trend = | 0.027 |  |  |  | P for trend = | 0.223 |
| **Hg** |  |  |  |  |  |  |  |  |  |  |
| Q1 (≤2.56) | 2,118 | 12 |  | 1.00 (referent) |  |  | 1,830 | 8 | 1.00 (referent) |  |
| Q2 (2.57-3.64) | 2,253 | 19 |  | 1.41  (0.68-2.92) | 0.350 |  | 1,734 | 10 | 1.30  (0.51-3.29) | 0.587 |
| Q3 (3.65-5.15) | 2,349 | 12 |  | 0.87  (0.39-1.94) | 0.730 |  | 1,655 | 7 | 0.94  (0.34-2.59) | 0.899 |
| Q4 (≥5.16) | 2,416 | 15 |  | 1.07  (0.50-2.30) | 0.862 |  | 1,574 | 7 | 0.97  (0.35-2.70) | 0.958 |
|  |  |  |  | P for trend = | 0.587 |  |  |  | P for trend = | 0.905 |
| **Se** |  |  |  |  |  |  |  |  |  |  |
| Q1 (≤157) | 2,335 | 17 |  | 1.00 (referent) |  |  | 1,626 | 10 | 1.00 (referent) |  |
| Q2 (158-168) | 2,099 | 14 |  | 0.92  (0.45-1.88) | 0.829 |  | 1,588 | 7 | 0.70  (0.27-1.86) | 0.478 |
| Q3 (169-182) | 2,349 | 12 |  | 0.70  (0.33-1.47) | 0.348 |  | 1,848 | 9 | 0.77  (0.31-1.91) | 0.579 |
| Q4 (≥183) | 2,353 | 15 |  | 0.89  (0.44-1.79) | 0.739 |  | 1,731 | 6 | 0.53  (0.19-1.48) | 0.226 |
|  |  |  |  | P for trend = | 0.810 |  |  |  | P for trend = | 0.670 |
| **Mn** |  |  |  |  |  |  |  |  |  |  |
| Q1 (≤12.5) | 2,107 | 13 |  | 1.00 (referent) |  |  | 1,751 | 8 | 1.00 (referent) |  |
| Q2 (12.6-15.2) | 2,273 | 15 |  | 1.11  (0.53-2.34) | 0.782 |  | 1,688 | 7 | 0.95  (0.34-2.65) | 0.928 |
| Q3 (15.3-18.6) | 2,401 | 11 |  | 0.79  (0.35-1.77) | 0.565 |  | 1,699 | 12 | 1.58  (0.64-3.89) | 0.319 |
| Q4 (≥18.7) | 2,355 | 19 |  | 1.40  (0.69-2.85) | 0.357 |  | 1,655 | 5 | 0.69  (0.22-2.11) | 0.511 |
|  |  |  |  | P for trend = | 0.488 |  |  |  | P for trend = | 0.412 |
| *P values were obtained from the multivariable logistic regression analysis adjusted for age, | | | | | | | | | |  |
| smoking habits of the partner, drinking habits, gravidity, parity, number of cesarean deliveries and geographic region. | | | | | | | | | |  |
